# Microbial Proteases as Product-Directed Catalysts for Industrial Proteolysis

**DOI:** 10.3390/biom16091317

**Published:** 2026-09-10

**Authors:** Sang-Woo Han

**Affiliations:** Department of Biotechnology, College of Biomedical & Health Science, Konkuk University, Chungju 27478, Chungbuk, Republic of Korea; swhan524@kku.ac.kr

**Keywords:** microbial protease, product-oriented proteolysis, protease engineering, peptide-pool remodeling, programmable proteolysis

## Abstract

Microbial proteases are widely used for industrial proteolysis in detergents, food processing, hydrolysate production, product stabilization, and side-stream valorization. Their industrial value is not determined by general activity or degree of hydrolysis alone, but by the ability of a defined catalyst to operate reliably under process conditions and generate the intended product endpoint. This review frames microbial proteases as product-directed biocatalysts whose performance depends on the alignment of catalyst identity, process compatibility, substrate access, and product outcome. Production, maturation, and immobilization are considered determinants of the deployed enzyme form, while biocatalyst identity, process translation, product outcome, and endpoint refinement provide a framework for evaluation. Across protease classes, industrial utility is shaped by acid-window matching, operating-window engineering, catalyst definition, metal-state compatibility, and peptide-pool refinement. Data-guided workflows integrating protease discovery and engineering, cleavage prediction, peptidomic mapping, real-substrate validation, and enzyme-system optimization offer a path toward more predictable product-oriented industrial proteolysis.

## 1. Introduction

Industrial proteolysis is central to detergent formulation, food processing, leather treatment, hydrolysate production, and valorization of protein-rich by-products. Proteases are major industrial enzymes because they can catalyze extensive protein degradation or selective peptide-bond cleavage under comparatively mild processing conditions, thereby replacing harsher chemical treatments in many applications [1,2]. Microbial proteases are especially important in this landscape because bacterial, fungal, archaeal, and metagenome-derived systems provide scalable production, catalytic diversity, and engineering flexibility [3]. These features make microbial proteases suitable not only for established enzyme markets, but also for emerging product-oriented processes where enzyme supply, catalyst form, and final product quality must be controlled together.

The industrial role of proteases is expanding beyond bulk protein degradation. In many classical uses, a protease preparation was judged mainly by whether it could degrade a protein efficiently under an approximate process condition. This remains important in some applications, but many current food and side-stream processes require a more specific interpretation of proteolysis. A hydrolysate is valuable when its peptide population has useful solubility, nutritional quality, sensory acceptability, bioactivity, or formulation behavior [4,5,6,7,8,9,10,11,12]. For example, a tenderizing or clotting enzyme is useful when it changes the target matrix while preserving product integrity [7,13,14,15,16]. A keratinase is valuable when it can open and convert insoluble and disulfide-rich substrates into recoverable products [17,18,19,20]. These examples show why industrial protease development is increasingly concerned with what product a protease creates, not only with whether the enzyme is active.

This shift changes how industrial proteases should be evaluated. Activity on casein, synthetic peptides, or other model substrates remains useful as an early descriptor, but it does not promise industrial performance. Model-substrate assays are often performed under simplified conditions that do not include detergent formulations, acidic beverage matrices, viscous protein slurries, or insoluble keratin-rich substrates [5,6,18,19,20,21,22,23,24]. Likewise, optimum pH and temperature provide useful biochemical information, but they do not indicate whether a catalyst survives formulation storage, remains active during operation, or produces a desired product. Degree of hydrolysis is also an incomplete descriptor. It reports the extent of peptide-bond cleavage, but not peptide identity, peptide sequence, molecular-weight distribution, or product functionality [4,5,6,9,10,11,12,25].

These descriptors, such as activity, stability, or degree of hydrolysis, become industrially meaningful only when they are connected to the catalytic form being utilized, the process environment in which the enzyme operates, the real substrate, and the intended final product. For example, a protease may show high apparent activity but fail in a detergent formulation because surfactants or storage conditions destabilize the enzyme [21,24]. A hydrolysate may reach a high degree of hydrolysis but still contain bitter peptides or weak bioactivity [4,5,9,10,11]. Product-oriented proteolysis therefore requires moving from isolated biochemical descriptors toward linked evidence connecting active catalyst form, process compatibility, substrate accessibility, and final product quality.

Recent reviews have summarized microbial protease classes and their industrial applications [2,3], while others have focused on food peptidase engineering or recombinant production strategies [1,26,27]. In contrast, this review considers microbial proteases from a product-directed perspective. It links the catalytic form of the enzyme to the process conditions in which it operates and to the final product to be generated. Accordingly, protease classes are discussed in terms of the design considerations associated with their application, rather than according to catalytic classification. This framework is further extended to data-guided protease selection and peptide-level validation. The main aim of this review is to show how microbial protease development can be organized around a desired product outcome.

**Figure 1:**
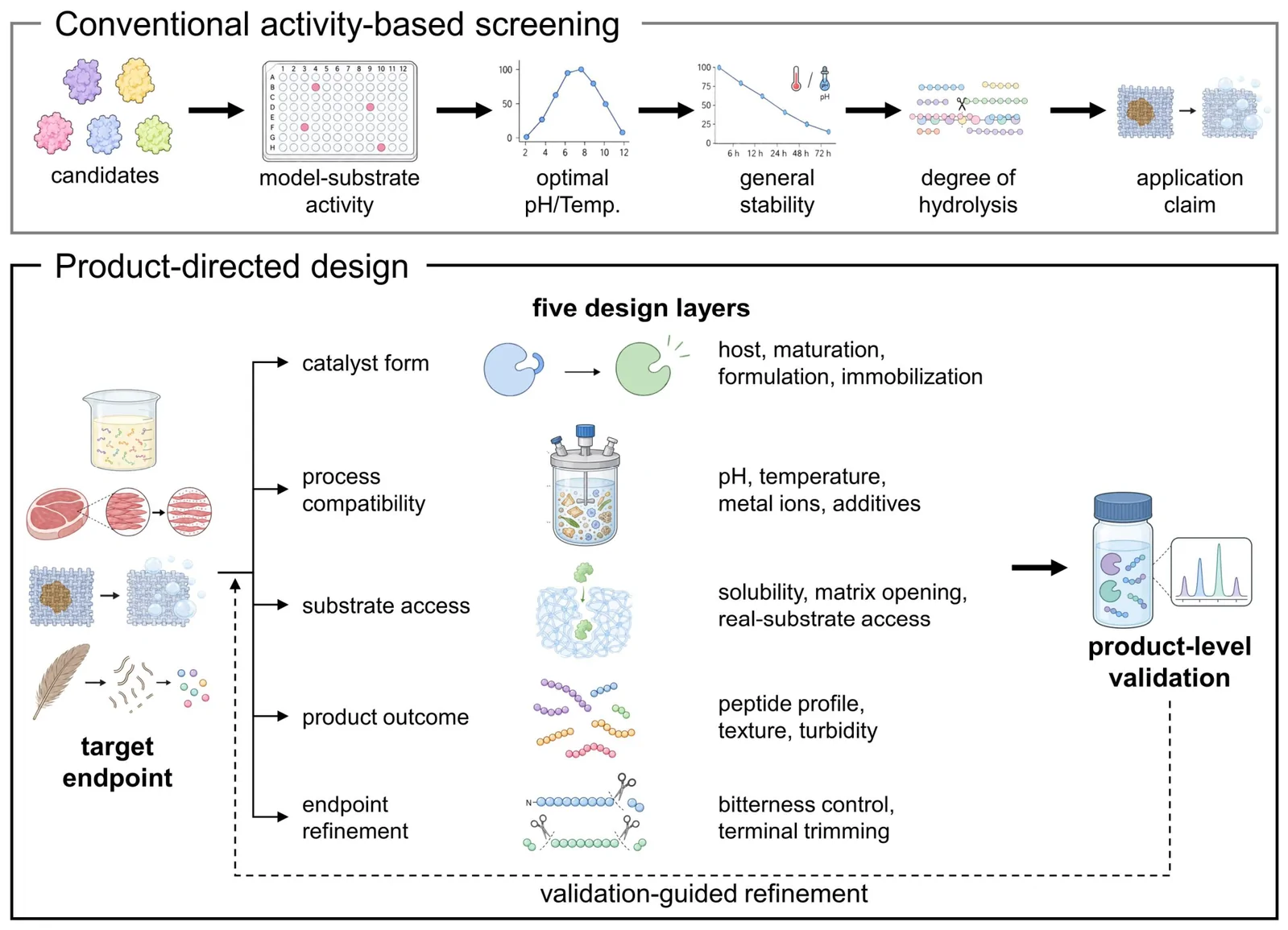
From activity-based screening to product-directed design of industrial microbial proteases. Conventional screening evaluates proteases mainly through general biochemical properties, which may not predict industrial product performance. Product-directed design instead aligns the deployed catalyst, process conditions, substrate accessibility, and cleavage outcome with a predefined product endpoint. Product-level validation then guides iterative refinement of the catalyst and process.

This product-oriented view is especially important because protease performance depends on the alignment of several design layers. Production and deployment format determine what catalyst enters the process. Biocatalyst identity determines whether the active molecular form is defined and reproducible. Process compatibility determines whether the catalyst retains operational robustness under practical conditions. Substrate fit determines whether the enzyme can access the relevant peptide bonds in the actual matrix. Product outcome determines whether the reaction produces the intended final product. A useful industrial protease is therefore the catalyst whose production form, operating window, substrate access, and cleavage outcome match the product being made. This shift from activity-centered evaluation to an alignment-based, product-directed design framework is summarized in Figure 1.

The following sections develop this argument by moving from industrial need to catalyst definition and then to endpoint-directed design. The review first identifies the application bottlenecks that determine whether a protease is useful, and then considers how production, maturation, and immobilization define the catalytic form that enters the process. This leads to an evaluation framework that separates biocatalyst identity, process translation, substrate fit, product outcome, and endpoint refinement. The class-specific sections then use aspartic, serine, cysteine, and metalloproteases as design cases showing how operating window, active-form control, engineering flexibility, and metal dependence shape industrial use. The final section extends this framework toward data-guided workflows in which cleavage prediction, peptidomic mapping, product-property modeling, real-substrate validation, and enzyme-system optimization are connected around a defined product endpoint.

## 2. Industrial Design Requirements for Product-Oriented Protease Applications

Microbial proteases are used in processes that differ in formulation chemistry, substrate accessibility, and product expectation. These differences mean that protease selection should begin with the intended industrial function rather than with protease type or apparent activity alone. A detergent protease, hydrolysate-producing protease, and meat-tenderizing enzyme are not judged by the same criteria because each application imposes a different bottleneck and endpoint readout [1,2,17]. Product-directed catalyst design therefore begins by identifying the dominant bottleneck of the process and then selecting, producing, or engineering a catalyst whose properties are relevant to that bottleneck. The major application bottlenecks, required protease roles, and corresponding product-level evidence are summarized in Table 1.

In formulation-driven applications, the key requirement is operational robustness. Detergent proteases must operate in chemically complex environments that combine alkaline pH, surfactants, and short reaction times on proteinaceous stains. A useful detergent enzyme must therefore retain catalytic activity under detergent-like conditions and contribute to physical stain removal, not merely show high activity in a simple buffer. Modern detergent protease studies are most persuasive when formulation tolerance and washing efficacy are reported together, as illustrated by engineered *Bacillus* proteases that link surfactant tolerance or formulation stability to application-level cleaning performance [21,24].

Hydrolysate production imposes a different requirement because the product is a peptide population. Degree of hydrolysis describes reaction progress, but it does not specify peptide sequence, molecular-weight distribution, or biological function. Industrial hydrolysates are valued as ingredients when their peptide populations provide useful antioxidant activity, nutritional quality, or acceptable taste. Enzyme selection should therefore be guided by the intended ingredient profile. Blue whiting hydrolysis, for example, shows that proteases applied to the same feedstock can generate hydrolysates with different product-level properties, separating reaction extent from product value [5].

Sensory quality and endpoint refinement add a further layer to hydrolysate design. A peptide mixture may be functional but commercially limited by bitterness, excessive heterogeneity, or an unfavorable balance of small peptides and free amino acids. This limitation cannot be solved by measuring total hydrolysis alone because sensory risk is shaped by sequence-specific peptide composition and terminal residues. In such cases, the primary endoprotease and the refinement enzyme should be treated as different catalytic roles. One generates the initial peptide pool, whereas the other remodels it toward the final product specification. Sequence-based tools such as BIPE (Bitterness Intensity Prediction Engine) can help flag bitterness risk, while aminopeptidase-mediated treatment provides a separate experimental route for endpoint correction [9,10].

Controlled structural modification requires limited proteolysis rather than extensive degradation. In meat tenderization and dairy coagulation, the enzyme must alter a protein matrix without destroying the structure that defines product quality. Insufficient proteolysis may fail to reduce toughness or promote clotting, whereas excessive proteolysis can lead to nonspecific matrix damage producing over-softened tissue or weak curd. These applications should therefore be evaluated by matrix-level outcomes such as shear force, clotting activity, curd behavior, and product integrity rather than by general protein degradation alone [7,13,14,16].

Selective stabilization and real-substrate valorization illustrate two additional application requirements. In stabilization-oriented proteolysis, the goal is to remove a quality-limiting protein fraction while preserving the broader product profile, as in chill-haze reduction without loss of foam or sensory quality [22]. In side-stream valorization, the challenge is often substrate access. Performance should therefore be demonstrated using real protein-rich wastes rather than inferred from soluble model substrates, as illustrated by microbial proteases tested across feathers, whey, and other industrial matrices [31]. For keratin-rich residues specifically, conversion and soluble-product (i.e., protein, peptides, or amino acids) recovery provide more relevant evidence than model-substrate activity [18].

Matrix selectivity becomes the dominant requirement when the target material is embedded within a valuable structure that must be preserved. In leather dehairing, hair keratin should be removed while collagen-rich hide remains intact. In structured foods, the target protein fraction should be modified without broad network collapse [15,32]. In these applications, stronger degradation can be less valuable than target selectivity. Product-oriented protease evaluation should therefore match the dominant bottleneck of each application to the corresponding product-level evidence, creating the basis for defining the catalytic form that must enter the process.

## 3. Production Platforms and Catalyst Definition

Production is not only a supply step in industrial protease development. It determines which catalytic form enters the process, how reproducibly that form is obtained, and whether the enzyme preparation can be linked to the intended product outcome. This issue is especially important for proteases because secretion, activation, and immobilization can alter enzyme orientation, active-site accessibility, and observed catalytic activity [1,7,26,33,34]. Two preparations with the same enzyme name may therefore differ in molecular form or enzyme properties. Production strategy should therefore be treated as part of biocatalyst definition rather than as a separate upstream step.

Native production remains useful when a crude preparation gives a robust and directly measurable application endpoint. Solid-state or submerged fermentation can be appropriate when preparation-level performance is the relevant industrial catalyst, as in some food-processing applications. A native *Mucor circinelloides* milk-clotting protease produced on dhal husk illustrates this application-oriented production logic [16]. Such cases are useful when the endpoint can be evaluated directly, but they should not be overinterpreted as evidence for a defined molecular catalyst unless the active species is also assigned.

Recombinant expression becomes more important when enzyme identity, engineering, or product reproducibility must be controlled. It can reduce background protease complexity and connect an application outcome to a specific molecular form. RmproA from *Rhizomucor miehei* illustrates this point because expression in *Pichia pastoris* generated a glycosylated enzyme form, as shown by the molecular-mass shift after Endo H treatment [7]. For precursor-dependent proteases, sequence discovery must also be followed by active-form verification. Globupain, for example, was produced as an inactive proenzyme and required reducing conditions, calcium, and autoprocessing to form the mature heterodimeric catalyst [34].

Secretion design and host choice provide additional layers of catalyst definition for extracellular proteases. Signal peptide choice affects secretion efficiency and extracellular recovery, whereas host physiology affects folding and protease tolerance. RmproB secretion in *Aspergillus niger* and GsProS8 recovery from *Bacillus subtilis* are useful examples because they connect production route to a recoverable catalyst that can be evaluated in later application contexts [14,35]. The best production platform is therefore the one that delivers the required active form reproducibly, not simply the one that gives the highest apparent activity.

Deployment format further changes catalyst behavior after production. Immobilization can alter enzyme orientation and active-site accessibility while improving operational stability and reusability [36]. Specific immobilized cysteine protease systems illustrate how these effects depend on the enzyme–support combination [33]. Immobilized, crude, and purified enzymes should therefore be treated as different catalyst formats rather than interchangeable versions of the same protease. This distinction is necessary because product-oriented claims should be tied to the exact catalyst format that contacts the substrate under the intended process conditions.

The main message of this section is that production should be integrated into protease evaluation. Native production may be sufficient when preparation-level performance is the industrial endpoint. Recombinant production becomes important when enzyme identity or engineering must be controlled. Host choice, secretion route, activation, and deployment format determine what catalyst is tested. In product-oriented industrial proteolysis, the enzyme is the catalytic form that enters the process and generates the intended final product.

## 4. Evaluation Framework from Biocatalyst Identity to Product Outcome

A product-oriented evaluation framework should ask three questions in order. First, what catalytic form is being tested? Second, can that form function under the intended process conditions? Third, does the reaction generate the intended product outcome? These questions separate biocatalyst identity, process translation, and endpoint design. Without this separation, a protease can appear promising in a model assay yet remain process-incompatible or unsuitable for the final product. The framework is therefore a way to prevent overinterpretation of activity measurements and to align experimental evidence with industrial claims.

The first layer is biocatalyst identity. This layer is fundamental because enzyme performance can be interpreted and reproduced only when the active catalyst entering the reaction is clearly defined. A preparation may show measurable activity while containing different proportions of partially processed forms, isoforms, or host-derived proteins. In such cases, enzyme loading may not represent the actual catalytic dose, and differences in process performance or product outcome cannot be attributed confidently to the intrinsic properties of a specific enzyme form. Active-form verification should therefore include the production host, maturation state, relevant post-translational modifications, and preparation type. Globupain illustrates this requirement because the expressed 52 kDa zymogen required reducing conditions (i.e., DTT), Ca^2+^ for activation, and autoprocessing to form the active heterodimer [34]. RmproA provides a complementary example, as expression in *P. pastoris* generated a glycosylated recombinant form identified by an Endo H-dependent molecular-mass shift [7]. These cases show that biocatalyst identity is not merely a reporting detail, but the basis for linking enzyme dose, process behavior, and product formation to the catalyst deployed.

The second layer is process translation. This layer is critical because high activity under ideal assay conditions has little industrial value if the catalyst is inhibited or loses stability during the actual process. Buffer-based assays establish catalytic competence, but do not show whether the enzyme can withstand formulation components, non-optimal pH, thermal exposure, or practical reaction times. Without process-relevant validation, promising laboratory activity can therefore lead to an overestimation of industrial performance. Evidence for process translation should consequently progress from retention of enzyme function under representative process stresses to demonstration of the intended application-level outcome. The N212S variant of *Bacillus clausii* bcPRO illustrates the first level by showing improved activity and retention in AES (Alkyl Ethoxy Sulfate) and LAS (Linear Alkylbenzene Sulfonate), indicating enhanced tolerance to detergent formulation stresses [24]. The A188P/V262I BAPB92 variant extends this evidence to the application level by linking improved enzyme properties to 17.84–18.51% higher washing efficacy in liquid detergent formulations [21]. These examples show that process translation is established most convincingly when biochemical performance is retained under relevant stresses and subsequently converted into the intended industrial outcome.

The third layer is endpoint design. This layer asks whether proteolysis generates the intended molecular and functional outcome. A reaction may reach high hydrolysis extent but still produce an undesirable product, such as a bitter hydrolysate and an epitope-containing peptide pool [9,10,12]. Endpoint evaluation should therefore be matched to the intended product property. Hydrolysate studies may require peptide-size distribution, peptide mapping, and bioactivity measurements [4,5,6,7,8,9,10,11,12], whereas structured-matrix applications require endpoint-specific measurements such as shear force, curd quality, or turbidity [7,13,14,15,16,22,23].

This framework can be operationalized as a reporting checklist (Table 2). A product-oriented protease study should report the active catalyst form, production or expression context, process-relevant assay conditions, product-level readout, and endpoint-specific limitation. Such reporting separates biochemical activity from industrial usefulness and makes studies easier to compare across substrates and applications. This framework defines how different types of evidence support different levels of industrial interpretation.

The following class sections apply this framework in different ways. Aspartic proteases emphasize acid-window endpoint matching. Serine proteases illustrate operating-window and bottleneck-specific engineering. Cysteine proteases highlight active-form definition and emerging designability. Metalloproteases show how metal-dependent catalysis can support peptide generation, peptide-pool remodeling, and process compatibility. Together, these classes show that industrial protease development is becoming less enzyme-centered and more product-directed.

## 5. Protease Classes as Distinct Design Solutions for Product-Oriented Proteolysis

### 5.1. Aspartic Proteases as Acid-Window Catalysts for Low-pH Product Transformation

Aspartic proteases are best framed as acid-window catalysts for low-pH product transformation. Their industrial value appears when acidic activity matches the process environment and helps generate a defined product outcome. This role is evident in applications such as milk clotting, acidic protein hydrolysis, and acidic beverage clarification. In these processes, the enzyme is useful not simply because it is active at low pH, but because its operating window fits an acidic process matrix. Acid-window proteases can therefore preserve product quality and support controlled cleavage under conditions compatible with the final product.

Milk clotting provides a useful case for evaluating acid-window proteolysis through a product-level endpoint. In this application, the enzyme should promote curd formation while avoiding excessive nonspecific proteolysis that compromises cheese quality. A native *M. circinelloides* milk-clotting protease produced by solid-state fermentation on dhal husk reached 7651 U/g activity and generated Cheddar cheese curd yield close to that obtained with commercial rennet [16]. This case shows that a native fungal protease preparation can be judged by a direct dairy endpoint, such as clotting performance and curd yield.

Recombinant aspartic proteases extend this logic by linking a defined enzyme form to specific product transformations. RmproA from *R. miehei* illustrates how an acid-window catalyst can support different endpoints depending on the substrate and process goal. In one application, recombinant RmproA produced in *P. pastoris* reduced pork shear force to 26.7 N, comparable to papain treatment under the tested conditions, indicating its potential for controlled meat tenderization [7]. In the same study, RmproA hydrolysis of turtle meat generated a large proportion of small peptides and achieved 88% ACE inhibition at 1 mg/mL hydrolysate. These outcomes are best interpreted as evidence that acidic proteolysis can be directed toward matrix softening or peptide generation when the operating pH, substrate, and endpoint are aligned.

Dairy coagulation provides a more selective version of the same design requirement. In this context, the useful enzyme must cleave casein in a way that supports clot formation while limiting nonspecific proteolysis that could weaken curd quality. RmproB illustrates this dairy-centered acid-window design. Recombinant secretion in *A. niger* produced an enzyme with high milk-clotting activity, a clotting activity/proteolytic activity ratio of 6.1, and κ-casein cleavage at sites including Phe105-Met106 [14]. This case connects acid protease performance to clotting selectivity and product-relevant cleavage behavior.

Acid-window proteases can also be used when the product-quality problem occurs in an acidic matrix. Beverage clarification is a useful example because the desired endpoint is turbidity reduction, not broad hydrolysate production. TlAP illustrates this application logic. The *Pichia*-expressed enzyme showed optimal activity at pH 3.0 and was applied to acidic fruit juice clarification, including apple juice, by reducing haze-forming proteins and turbidity [23]. This study broadens the role of aspartic proteases beyond dairy and protein hydrolysates, showing that an acidic protease can function as a selective product-stabilization tool when its pH window matches the beverage matrix.

Together, these examples support a restrained but useful interpretation of aspartic proteases in industrial proteolysis. Their current application value is best explained by acid-window endpoint matching. Future studies should still report mature-form and activation-state information when precursor processing affects catalyst identity, but the current application evidence most strongly supports acid-window matching between enzyme, process matrix, and final product.

### 5.2. Serine Proteases as Operating-Window-Engineered Workhorse Platforms

Serine proteases provide a strong model for process-matched protease engineering. For *Bacillus*-derived subtilisin-like enzymes and microbial keratinases, the key question is no longer whether the enzyme can hydrolyze protein. The more relevant question is which industrial bottleneck can be engineered. Depending on the application, this bottleneck may be thermal stability, formulation tolerance, real-substrate conversion, or matrix selectivity. This makes serine proteases useful examples of the broader shift from activity improvement toward process-specific catalyst design. Their value lies not only in catalytic robustness, but also in the ability to connect engineering strategies to practical process requirements.

Low-temperature operation illustrates how serine protease engineering can expand an enzyme’s usable process window. Cold washing, low-temperature food processing, and energy-saving operations require enzymes that show considerable performance under conditions where many proteases lose catalytic efficiency. However, cold activity often involves a trade-off, because increased local flexibility can improve catalysis at low temperature while reducing structural robustness. Engineering therefore needs to enhance low-temperature activity without sacrificing stability. This requirement has been addressed through structure-guided redesign of *B. clausii* bcPRO. The MT6 variant increased catalytic efficiency at 10 °C from 0.11 to 2.08 μM^−1^ s^−1^ by modifying substrate-tunnel entrance loops [44]. DHAP variants provide a complementary example, with P9S/K27Q and P9S/T162I showing approximately five-fold higher caseinolytic activity at 15 °C than the wild type [45].

Thermostability addresses the opposite operating constraint. In high-temperature hydrolysis, a protease may benefit from faster reaction rates and increased substrate solubility, but only if it remains active long enough during the process. Thus, thermal performance should be evaluated by thermal stability rather than by optimum temperature alone. This design logic is illustrated by the AprE 2709 M14 variant, which extended the half-life at 60 °C from 19 to 74 min and increased residual activity after 60 min at 60 °C from 0.39% to 63.81% [46].

Detergent applications require another type of operating-window engineering because the enzyme must function in a chemically complex formulation. Detergent proteases must tolerate formulation chemistry, including alkaline pH and surfactants, as well as operating conditions such as dilution during washing and short contact times with proteinaceous stains. Therefore, engineering should be evaluated under detergent-like stress and, ideally, by washing performance. The A188P/V262I BAPB92 variant is the main application-level example because it connected enzyme engineering to an improvement of 17.84–18.51% in washing efficacy in detergent formulations [21]. The N212S variant of *B. clausii* bcPRO provides a supporting formulation-stress example by improving activity in AES and LAS and retained 46% residual activity after 4 h in LAS, compared with 15% for the wild-type enzyme [24].

Keratinase engineering highlights a different bottleneck, namely conversion of a recalcitrant substrate. In this section, the emphasis is placed on engineering strategies rather than on reusing the same feather-fermentation anchor cases introduced elsewhere. KerSMD truncation shows how domain architecture can be tuned for process robustness. Removal of the C-terminal PPC domain produced variants with improved catalytic efficiency and tolerance, including a V355 variant with a *k*_cat_/*K*_M_ of 143.6 s^−1^ mM^−1^ toward an AAPF synthetic peptide and more than 40% activity improvement under harsh conditions such as 15% NaCl or 4% SDS [29]. Active-pocket redesign provides a second route. The G209F/G235S mutant of KerBv doubled keratinolytic activity from 4270 to 8540 U/mL, increased the keratin-to-casein hydrolytic ratio to 1.64, enlarged the active pocket from 561 to 689 Å^3^, and improved feather degradation from 55.4% to 83.5% under the tested conditions [30]. These examples show how serine keratinase engineering can target active-site access and real-substrate conversion without relying only on soluble model-substrate activity.

Serine protease engineering can also be directed toward product composition. In hydrolysate and bioactive peptide applications, the important question is which peptides are produced. This shifts the engineering target from activity or stability toward cleavage outcome. The S166E variant of protease SE5 illustrates this product-composition logic. The variant increased sericin hydrolysis efficiency from 83.8 to 140.0 μg peptide/mL and increased ACE inhibition from 29.5% to 40.2% [4]. LC-MS/MS also identified unique peptides in the S166E hydrolysate, including HHSGVNR and GWWWNSD, that were not detected in the wild-type hydrolysate. This case shows that protein engineering can alter the peptide pool, not only the extent of hydrolysis. Product-directed serine protease engineering should therefore consider cleavage profile and endpoint activity alongside conventional activity metrics.

Matrix selectivity is another product-sensitive engineering target. In leather dehairing, excessive collagen degradation can damage the product even when keratin removal is efficient. KerZ1 S100D/Y208V illustrates this selectivity requirement. This variant preserved keratinase activity related to dehairing performance while reducing collagenase activity by 60% [32].

Overall, serine proteases are best presented as operating-window-engineered workhorse platforms. Their development illustrates how protease engineering can be aligned with three major industrial targets: operating-window expansion, difficult-substrate conversion, and product-sensitive cleavage control. The broader lesson is that serine protease engineering becomes industrially meaningful when the engineered property is explicitly connected to the process condition and product outcome it is intended to improve.

### 5.3. Cysteine Proteases from Source-Derived Catalytic Power to Defined and Designable Catalysts

Cysteine proteases occupy an important transition point in industrial proteolysis. Plant-derived cysteine proteases are already widely used under mild processing conditions. However, such source-derived preparations can vary in isoform composition and activity profile [47]. This creates a design challenge for product-oriented proteolysis, where reproducible peptide profiles and controlled endpoint formation may be required.

This challenge motivates interest in microbial, metagenomic, and designed cysteine proteases. The aim is not to replace established source-derived preparations, but to retain the practical catalytic advantages of cysteine proteolysis while improving reproducibility and engineering control. More defined systems become valuable when the product depends on specific cleavage behavior or predictable peptide-pool composition. In this sense, cysteine protease development is moving from broad catalytic utility toward catalyst-defined deployment. Application-oriented microbial protease reports show industrial promise, but they do not by themselves establish a defined catalytic species [48]. The useful message for product-oriented proteolysis is therefore that microbial and metagenomic systems create opportunities to move toward purified and reproducible catalysts.

A more defined route is illustrated by Globupain, a metagenome-derived C11 cysteine protease. For precursor-dependent proteases, identifying the sequence is only the first step because the translated enzyme may initially be an inactive zymogen. Globupain was produced as a 52 kDa inactive zymogen and required DTT/Ca^2+^ for activation, followed by autoprocessing into a mature heterodimer composed of 32 kDa and 12 kDa chains [34]. This case shows how cysteine protease discovery can be connected to active form verification.

Computational design extends this trajectory beyond natural discovery. A recent preprint used RFD2-MI to design cysteine proteases containing a Cys-His-Asp catalytic triad and a peptide-binding cleft for sequence-dependent peptide-bond hydrolysis [49]. Of 69 designs tested, 13 showed cleavage activity, and LC-MS confirmed cleavage at the intended target site. Further optimization produced Dokki_15, which showed an approximately 3 × 10^7^-fold rate enhancement over uncatalyzed peptide-bond hydrolysis. It shows that cysteine protease activity can now be built toward selected peptide-bond transformations, but industrial deployment will still require active-form stability, process compatibility, and product-level validation.

Taken together, these examples define the current direction of cysteine protease development. The practical use of cysteine proteases is well established, but product-directed proteolysis requires better catalyst definition. Globupain shows how metagenomic discovery can be linked to a verified active catalyst. Computational design suggests that future cysteine proteases may be built rather than only sourced. The central challenge is now to connect catalytic discovery or design to microbial production, active-form control, and reproducible product outcomes.

### 5.4. Metalloproteases as Metal-Dependent Catalysts for Peptide Generation and Peptide-Pool Remodeling

Metalloproteases should be organized by industrial role rather than by class label alone. Their common feature is metal-dependent catalysis, but their industrial functions can differ substantially. Some metalloproteases act as endoproteases that contribute to primary peptide generation, whereas others act as exopeptidases that refine peptide termini after hydrolysis. In both cases, the metal state of the enzyme is not a minor biochemical detail. Metalloproteases encompass functionally diverse catalysts rather than a single type of broadly degradative enzyme [50]. In industrial systems, some act as endoproteases for primary peptide generation, whereas others function as exopeptidases for terminal trimming [8,9,39]. Because chelators and competing ions can alter the catalytic metal state, metal compatibility should be evaluated under the intended process conditions [40]. Metalloprotease evaluation should therefore ask whether the enzyme remains functional in the metal and matrix environment of the intended process.

The first role is primary peptide generation. This role is important because metalloproteases are sometimes discussed mainly as terminal-trimming or refinement enzymes, but neutral metalloproteases can also participate in endoproteolytic hydrolysis that shapes the initial peptide pool. Thermolysin-like metalloproteases provide this broader context. Thermolysin hydrolysis of heat-denatured β-lactoglobulin exposed cleavage sites that were less accessible in the native protein and released LQKW, a previously identified ACE-inhibitory peptide [51]. This example shows how process-induced changes in substrate accessibility can influence the primary peptide pool generated by a metalloprotease. A thermostable neutral metalloprotease from *Geobacillus* sp. EA1 provides a complementary specificity-focused example. PICS-based substrate profiling and mutational analysis showed that EA1 does not simply reproduce thermolysin’s strict P1′ leucine preference but instead has a more flexible S1′ pocket that broadens its potential cleavage range [39]. These cases support the idea that metalloproteases can shape primary peptide pools through both process-compatible stability and distinctive substrate preference.

The clearest product-oriented role for this class is endpoint refinement after primary hydrolysis. Once an endoprotease has generated a peptide mixture, product quality may still be limited by bitterness, excessive heterogeneity, or an unfavorable peptide-size distribution. Metal-dependent aminopeptidases can address this problem by trimming peptide termini and remodeling the peptide pool. ScAP from *Streptomyces canus* T20 illustrates aminopeptidase-based endpoint refinement because optimized treatment in rice peptide preparation increased small-peptide formation and reduced bitter intensity by 49.0% [9]. This case shows that metalloprotease-type exopeptidases should be interpreted as endpoint-refinement enzymes rather than as the primary proteases responsible for bulk hydrolysis.

The final requirement is metal-state compatibility. A metalloprotease that performs well in a defined buffer may behave differently in practical process matrices, where chelators, competing ions, and formulation components can alter metal availability. ChtCP from *Chitinophaga* illustrates why this variable should be reported explicitly. ChtCP retained activity at high temperature, but EDTA abolished activity, while Co^2+^ increased its melting temperature by 25.3 °C [40]. This case links metalloprotease performance to the ionic environment. For industrial use, metal compatibility is therefore part of process translation, not an optional characterization detail.

Metalloproteases are best treated as metal-sensitive catalysts whose industrial value depends on role and context. This framing makes the section broader than terminal peptide trimming while keeping the claims aligned with the available evidence. Metalloprotease studies should therefore report substrate conversion together with metal state and product-level outcomes. Across the four protease classes, the distinct design logics, product-oriented roles, and class-specific evaluation checkpoints are compared in Table 3.

## 6. Product-Oriented Applications Across Protease Classes

Product-oriented applications can be reorganized by endpoint rather than by protease class. The useful question is how different catalytic classes and cleavage behaviors solve different parts of the same product problem. A hydrolysate process may require broad endoproteolysis and terminal trimming. A structured food process may require limited matrix modification, and a side-stream process may require substrate opening before peptide-bond cleavage. Protease selection should be based on the defined role of each catalyst within a product workflow.

### 6.1. Hydrolysate Design Through Protease Selection and Combination

Functional hydrolysate and bioactive peptide production illustrate this cross-class logic well. Serine proteases can support rapid primary hydrolysis under neutral-to-alkaline conditions, whereas aspartic proteases are useful when the substrate or process favors an acidic environment. Neutral metalloproteases can also generate primary peptide pools, while aminopeptidases and mixed endo-/exopeptidase systems can refine peptides after initial cleavage. Comparative studies show why such catalytic complementarity matters. In blue whiting hydrolysis, Alcalase, Savinase, Neutrase, papain, Protamex, and Flavourzyme were applied separately, and each preparation generated a different balance of recovery, antioxidant activity, and beverage compatibility [5]. Likewise, separate treatment of poultry by-products with bromelain or Endocut-02 produced different molecular-weight distributions and hydrolysate profiles [6]. These studies show that individual proteases generate different starting peptide pools. Therefore, protease complementarity should be considered not only in terms of total hydrolysis, but also through differences in cleavage breadth and peptide-size distribution [52].

Commercial proteases also illustrate how enzymes with complementary catalytic roles can be combined within practical hydrolysis processes [5,6]. In poultry meal hydrolysis, sequential Alcalase–Flavourzyme treatment gave higher hydrolysis and product recovery than simultaneous treatment or either enzyme alone [11]. A broader multienzyme composition containing Alcalase, Neutrase, Flavourzyme, and Protamex was also developed for poultry-processing residues and optimized toward protein recovery and product properties such as antigenicity and bitterness [53]. These studies show that commercial proteases can be combined according to complementary catalytic roles rather than used only as individual catalysts.

A recent study further shows that the benefit of multienzyme systems depends on both enzyme combination and reaction sequence. In soybean meal hydrolysis, Alcalase, Protamex, papain, or trypsin was combined with Flavourzyme and applied by simultaneous or sequential treatment [37]. Sequential hydrolysis generally produced lower bitterness than simultaneous treatment, while the Protamex–Flavourzyme combination showed the best balance between debittering and functional improvement under the tested conditions. Peptide analysis provided a molecular basis for this effect. Flavourzyme reduced the enrichment of N-terminal Val, Ile, and Leu in peptides generated by Protamex, indicating that terminal trimming changed the peptide population associated with bitterness. Therefore, multienzyme design should consider both enzyme specificity and reaction sequence, particularly when the target product is defined by peptide composition rather than by the overall extent of hydrolysis.

However, multienzyme systems also require careful process control. Individual enzymes may have different operating conditions, and the optimal condition for one enzyme may not be suitable for another. Enzyme ratio and reaction time should be optimized for the complete system rather than for each enzyme separately. The key design question is how complementary enzymes should be selected, combined, and ordered to generate the intended hydrolysate profile.

### 6.2. Endpoint Control for Sensory Quality and Antigenicity

Sensory improvement and antigenicity reduction require more specific endpoint evidence. In low-bitterness hydrolysates, the limiting factor is the persistence of sequence-specific bitter peptides and terminal residues [9,10,11]. The multienzyme studies discussed above show that enzyme order and terminal trimming can change the peptide population and reduce bitterness. Endpoint evaluation should therefore include peptide-level information or sensory measurements.

In hypoallergenic products, the relevant endpoint is the removal of epitope-containing peptides. In whey protein hydrolysis, bioinformatics-guided protease selection and peptide mass fingerprinting linked reduced antigenicity to the removal of longer epitope-containing peptides. In targeted whey protein hydrolysis, Alcalase and papain were identified as effective enzymes for degrading α-lactalbumin and β-lactoglobulin and reducing residual IgG binding among the five proteases tested [12].

### 6.3. Selective Proteolysis for Texture and Product Stabilization

Texture-directed and stabilization-oriented proteolysis represent applications where limited and selective cleavage is more important than extensive hydrolysis. In meat tenderization, broad cysteine protease preparations have practical utility, but more defined microbial proteases may offer better control when avoidance of over-softening is required. A *B. subtilis* alkaline protease reduced carabeef toughness by approximately 80%, providing an application-level example of microbial protease-based tenderization evaluated by shear force [13]. Collagenolytic proteases from *B. subtilis* B13 and *Bacillus siamensis* S6 add a more selective comparison because they showed preferential collagenolytic activity with relatively low myofibrillar protein hydrolysis, matching the need to weaken connective tissue while preserving broader meat structure [15]. In beverage stabilization, *A. niger* prolyl endoprotease reduced chill haze and maintained turbidity below 1 EBC after 6 months while preserving foam stability and higher polyphenol content than PVPP treatment [22]. Across these endpoints, the relevant comparison is the balance between target modification and product preservation.

### 6.4. Protease Systems for Side-Stream Conversion and Matrix Preservation

Side-stream and matrix-sensitive applications add the requirement of substrate access and non-target preservation. In this application-level section, the protease–reductase chimera is the most useful example because it shows that an effective catalyst system may need a substrate-opening function in addition to peptide-bond cleavage. Coupling disulfide reduction with proteolysis increased feather protein release two-fold and free amino acid release 8.4-fold from keratin-rich material [20]. When the target substrate is embedded in a valuable matrix, such as hair keratin in collagen-rich hide, selectivity becomes the decisive criterion. This principle is consistent with engineered keratinase selectivity, where reducing collagenase activity while preserving dehairing performance improves matrix-sensitive processing [32].

These application areas show why product-oriented proteolysis should be endpoint-led and catalyst-role-specific. Across endpoints, the most useful protease is not necessarily the most active enzyme, but the catalyst whose operating window, substrate access, cleavage behavior, and product outcome match the intended final product. This synthesis also explains why data-guided workflows are needed. Once the desired endpoint is defined, protease choice, cleavage prediction, peptide mapping, and enzyme-system design can be organized around the product that must be generated.

## 7. Toward Data-Guided Workflows for Programmable Proteolysis

**Figure 2:**
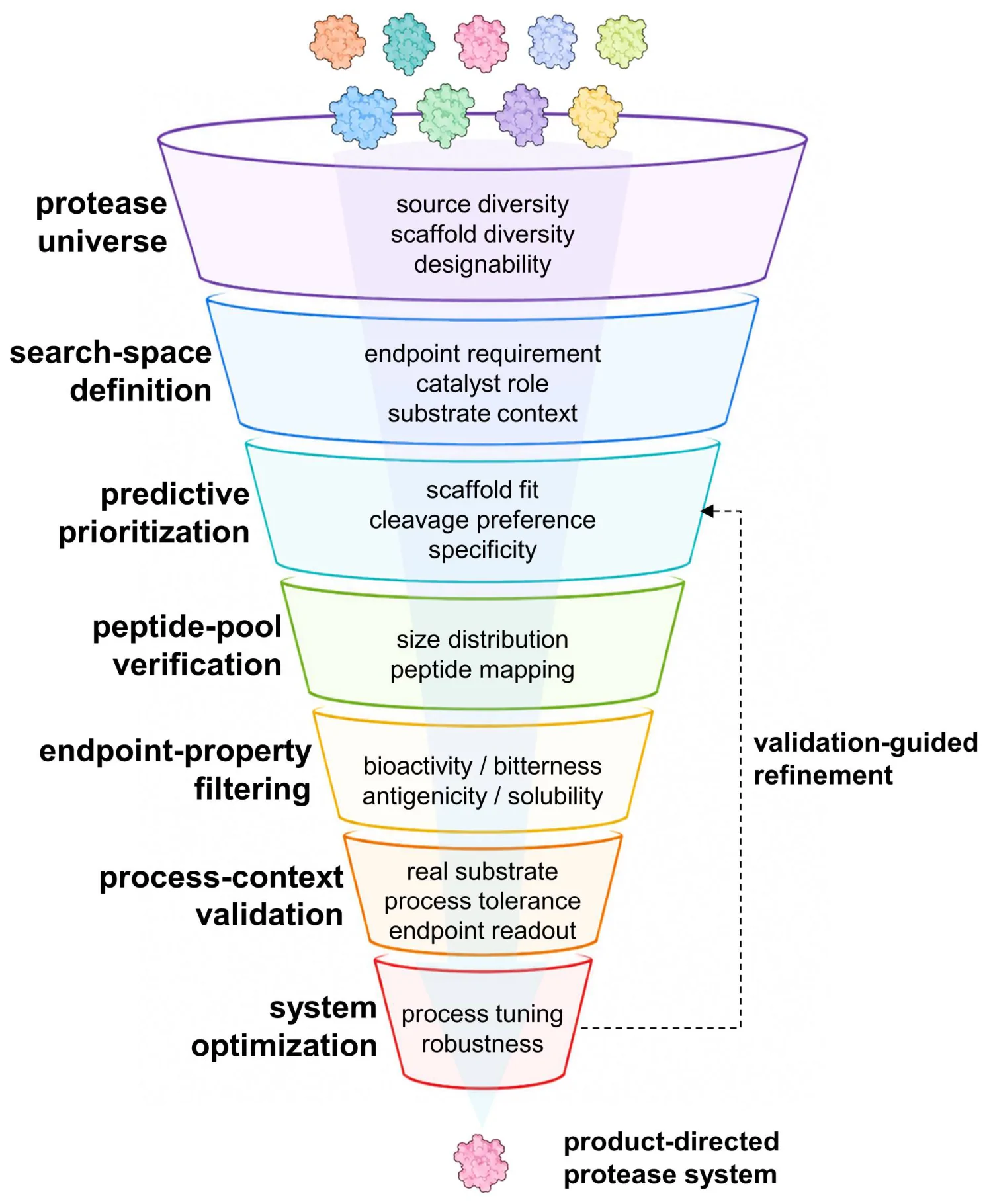
Data-guided workflow for endpoint-directed protease development. The workflow narrows a broad protease search space according to the intended product endpoint and uses computational prioritization, peptide-level analysis, and real-substrate validation to identify suitable catalysts. Experimental results are subsequently used to optimize enzyme combinations, reaction conditions, and catalyst robustness, creating an iterative path from candidate selection to a product-directed protease system.

Endpoint-directed proteolysis requires more than screening many enzymes against one substrate. The workflow proposed here is not a routine industrial pipeline that has already been fully established. Rather, it integrates capabilities that are emerging separately across protease research, including enzyme discovery, computational prediction, robustness engineering, and multi-enzyme system design. The goal is to connect these components into iterative product-directed development cycles in which each experiment answers a defined product question. The integrated sequence of endpoint definition, catalyst search, cleavage prioritization, peptide mapping, real-substrate validation, and iterative system refinement is summarized in Figure 2.

### 7.1. Endpoint Definition and Catalyst Prioritization

The first step is to define the product endpoint before enzyme selection. A bioactive peptide process requires functional validation, an antigenicity-reduction process requires immunological readouts, and a low-bitterness hydrolysate requires sensory-risk assessment. Texture-directed, stabilization-oriented, and side-stream processes add matrix-level requirements such as tenderness, turbidity, or substrate conversion. Once the endpoint is defined, candidate proteases should be assigned by catalytic role. Candidate enzymes may function as acid-window catalysts, primary endoproteases, endpoint-refinement enzymes, or matrix-selective catalysts.

A data-guided workflow can begin by expanding the catalyst search space. Structure-based search tools such as Foldseek make it possible to compare predicted or experimentally determined protein structures at large scale and may help identify distant protease scaffolds that would be missed by sequence homology alone [54]. In the context of industrial proteolysis, such tools are most useful for hypothesis generation. Those tools can broaden the set of candidate scaffolds, but they do not establish product-level performance.

### 7.2. Cleavage Prediction and Specificity Profiling

Cleavage profiling and computational prediction can then be used to prioritize hypotheses before extensive experimental testing. Sequence-based tools such as iProt-Sub [55], DeepCleave [56], and MPCutter [57] estimate cleavage-site preferences from sequence information, whereas CleaveNet [58] and PGCN [41] represent a more design-oriented layer that learns protease-substrate constraints or structure-aware specificity. CleaveNet illustrates this stronger design logic because it used deep learning architectures to learn protease-substrate constraints and design orthogonal peptide substrates for targeted proteases [58]. More recent protein language model and graph-based approaches extend this layer by learning sequence-context or structural features that may help rank candidate cleavage events before experiments are performed [59]. Prediction can also be coupled directly to high-throughput sequence–activity measurements. A DNA-recording platform evaluated approximately 600,000 protease–substrate pairs and used an epistasis-aware machine-learning model to identify protease variants with defined on- and off-target activity profiles [42]. This approach illustrates how experimental specificity data can be converted into iterative catalyst-design rules.

**Figure 3:**
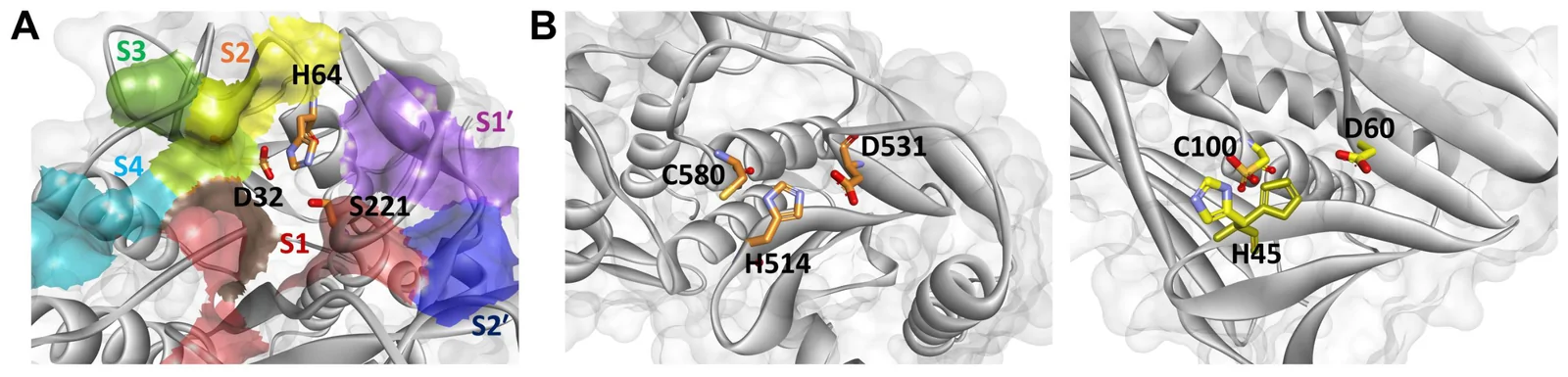
Representative structural frameworks for protease engineering and de novo design. (**A**) Substrate-recognition region of subtilisin Carlsberg (PDB ID: 1C3L), representing a serine protease scaffold. The catalytic triad D32–H64–S221 is shown together with the non-prime-side S1–S4 subsites and the prime-side S1′–S2′ recognition regions. (**B**) Comparison of the catalytic centers of the natural cysteine protease Ulp1 (PDB ID: 1EUV, left; catalytic triad C580–H514–D531) and the de novo-designed cysteine protease Dokki_15 (PDB ID: 9YNM, right), illustrating the construction of a Cys–His–Asp catalytic geometry within a newly designed protein scaffold.

Structure-guided engineering provides a complementary route for converting predicted or experimentally identified determinants into testable protease variants (Figure 3). In established protease scaffolds, engineering can target substrate-recognition regions or structural features that control catalytic performance. Subtilisin-like serine proteases provide a representative example, in which the catalytic center is surrounded by multiple substrate-binding subsites that offer targets for specificity engineering. In *Mucor pusillus* pepsin, docking-guided analysis of κ-casein was combined with site-directed mutagenesis of substrate-binding regions. The T218S mutation in the S2 region and L287G mutation in the S4 region altered the balance between milk-clotting and general proteolytic activity, with T218S increasing the clotting-to-proteolytic activity ratio by 3.34-fold [60]. A different structural mechanism is illustrated by the microbial cysteine protease SpeB. Structural and mutational analysis showed that its glycine-rich C-terminal loop controls active-site accessibility, with G378A favoring an occluded state and G385A favoring an open state that increased *k*_cat_ approximately 3.1-fold [61]. In metalloproteases, modification of the S1′ substrate-recognition pocket provides another route to cleavage control. In the *Geobacillus* sp. EA1 neutral protease, the F133L substitution shifted P1′ substrate preference toward that of thermolysin [39]. These examples show that structure-guided engineering can modify substrate recognition or active-site accessibility without directly changing catalytic residues.

Structure-guided design can also extend beyond modification of natural protease scaffolds (Figure 3B). Recent de novo cysteine protease design demonstrates that catalytic geometry and peptide-binding environments can be constructed within newly designed protein folds [49]. The Dokki protease study demonstrated sequence-dependent peptide-bond cleavage and provides a complementary example of how computational design can expand the engineering space beyond naturally occurring protease scaffolds. Such designs still require validation of active-form stability, process compatibility, and product-level performance before they can be translated toward product-directed proteolysis.

Experimental specificity profiling provides a complementary source of evidence because it measures what a protease cleaves under defined assay conditions. Proteome-derived peptide libraries, exemplified by PICS, can resolve prime- and non-prime-side preferences and subsite cooperativity from large sets of experimentally observed cleavage sequences [62]. Profiling methods and high-throughput engineering platforms are useful for mapping subsite preferences, identifying sequence specificity, and generating variants with altered cleavage behavior [63]. This information can guide enzyme choice or engineering before real-substrate testing, but the claim should remain calibrated to the assay. Specificity measured on peptide libraries or soluble reporters supports cleavage preference, whereas industrial performance still requires validation in the relevant matrix.

### 7.3. Peptide Mapping and Product Interpretation

After candidate cleavage hypotheses are prioritized, peptidomic product mapping is necessary to determine which peptides accumulate. Predicted cleavage events and transient fragments may not correspond to the final peptide population that remains under process conditions. Peptide mapping therefore connects catalyst action to product interpretation. In antigenicity-oriented whey protein hydrolysis, peptide mass fingerprinting was used to identify epitope-containing peptides that persisted after proteolysis and were associated with residual IgG binding [12].

The peptide map must then be translated into product meaning. Time-resolved peptide mapping can also guide reaction termination rather than only characterize the final hydrolysate. During micellar-casein hydrolysis, a sensopeptidomic analysis identified 22 peptides strongly associated with bitterness and indicated a 5 h stopping point that provided the best sensory balance under the tested conditions [38]. Product-property prediction can help interpret large peptide datasets when the endpoint depends on sequence-level features. BIPE provides one example by estimating peptide bitterness intensity from peptide sequence information using a protein language model-based regression approach [10]. In this workflow, BIPE would be used to flag peptide sequences that may contribute critically to bitterness, but not to identify the enzyme required for debittering. Beyond identifying undesirable peptide attributes such as bitterness, peptide-level prediction can also prioritize beneficial product features. Peptide_MDI illustrates this broader use by narrowing 2798 wheat-gliadin-derived candidates to a CaSR-binding lead peptide with experimentally validated affinity and cell-based antioxidant effects [64].

### 7.4. Real-Substrate Validation and Enzyme-System Optimization

Real-substrate validation should then test whether model-based hypotheses remain meaningful in complex matrices. KER0199 from *Amycolatopsis* sp. BJA-103 provides a useful example, not as a prediction-screening case, but as a study combining feather-powder activity with AlphaFold3/MD-based interpretation of keratin binding. Its increased activity toward feather powder in the presence of reducing agents further emphasizes that keratin conversion depends on substrate accessibility as well as enzyme-substrate recognition [19]. This type of evidence helps connect substrate accessibility, enzyme-substrate interaction, and process-relevant conversion.

The enzyme system should then be refined around endpoint performance. Targeted industrial proteolysis often requires systematically designed reaction schemes, either to open recalcitrant substrates before hydrolysis or to refine intermediate hydrolysates after primary cleavage. Product-directed workflows therefore benefit from stepwise or deliberately combined enzyme systems in which each catalytic component has an assigned role. Stepwise dual-enzyme hydrolysis of bovine bone protein illustrates why enzyme order can change hydrolysis outcome. In this study, trypsin followed by neutral protease achieved an overall degree of hydrolysis of 18.34%, exceeding simultaneous dual-enzyme hydrolysis at 13.81% and single-enzyme treatments such as neutral protease alone at 8.61% [65]. Dual-enzyme hydrolysis of *Pleurotus pulmonarius* powder further illustrates how data-guided optimization can tune process conditions after the enzyme system has been selected. In this case, an ANN (Artificial Neural Network) model predicted nitrogen recovery from hydrolysis variables, and a genetic algorithm searched the model space to identify optimal conditions of 50.9 °C, 6.45 h, and pH 6.12, yielding 27.23% nitrogen recovery with lower prediction error than response-surface modeling [43].

A final layer is robustness engineering, because a computationally selected protease is useful industrially only if it retains performance under process stress. ProteinMPNN-based redesign has been used to improve stability and activity while preserving key active-site constraints, whereas combined computational design of boiling-resistant keratinase illustrates how such tools can be connected to operating-window expansion [66,67].

### 7.5. Industrial Translation and Scale-Up Challenges

Industrial translation introduces additional constraints beyond laboratory-scale performance. A protease that performs well in a small-scale assay must also be produced in a reproducible form and used at a practical enzyme loading. During larger-scale fermentation, factors such as oxygen transfer and secretion efficiency can affect enzyme yield, while downstream recovery can increase production cost [68]. These changes may also affect the catalyst preparation that enters the final process. Therefore, scale-up should maintain enzyme productivity and the catalyst state required for the intended product outcome.

Process control is also important during scale-up. Immobilization or enzyme reuse can reduce catalyst demand when sufficient operational stability is maintained [36]. Reaction monitoring is also needed when product quality depends on a narrow range of proteolysis. In such cases, endpoint-related measurements can provide more useful process information than total protease activity. Consequently, scale-up should preserve the link between catalyst state, reaction conditions, and the intended product endpoint.

The proposed workflow is therefore iterative and extends from catalyst prioritization to industrial translation. Digital tools first help prioritize candidate proteases, while product mapping connects cleavage behavior with product meaning. Real-substrate validation and process optimization then test whether the selected system remains effective under practical conditions. Industrial translation further requires reproducible catalyst production and control of the product endpoint during scale-up. The next stage in industrial protease development is not simply to screen more enzymes, but to connect digital prediction, process validation, and product-level evidence around a defined product endpoint.

## 8. Conclusions

Microbial proteases remain essential industrial catalysts, but their future value will not be defined by broad activity alone. The field is moving toward product-directed catalyst design, where enzyme production, process compatibility, and product outcome are evaluated together. Aspartic proteases illustrate acid-window endpoint matching, serine proteases show operating-window and bottleneck-specific engineering, cysteine proteases show the importance of active-form definition and emerging designability, and metalloproteases show how metal-dependent enzymes can contribute to peptide generation, peptide-pool refinement, and metal-state-dependent process compatibility.

The broader implication is that industrial protease development should begin with the product question. A detergent process asks for formulation robustness, a hydrolysate process asks for peptide-pool quality, a structural process asks for controlled matrix transformation, and a side-stream process asks for real-substrate valorization. This shift also changes how protease studies should be reported, because activity data become most useful when linked to biocatalyst identity, real-substrate performance, and product outcome.

Data-guided workflows can make these choices more rational by linking endpoint definition, catalyst role assignment, functional prediction, enzyme-system optimization, robustness engineering, and industrial translation. Such workflows should not be viewed as replacements for experimental validation, but as ways to make validation more targeted and interpretable. The next useful industrial protease will be selected not because it is broadly active, but because its operating window and cleavage outcome are deliberately matched to the final product being made.

## Figures and Tables

**Table 1:** Application bottlenecks and product-level evidence in product-oriented industrial proteolysis discussed in this review.

Endpoint Product or Application	Dominant Bottleneck	Required Protease Role	Product-Level Evidence	Ref.
Detergent stain removal	Formulation stress; short-contact operation	Formulation-robust alkaline protease	Surfactant tolerance; residual activity; washing efficacy; storage stability	[21,24,28]
Functional protein hydrolysates	Peptide-profile generation; ingredient functionality	Primary endoprotease; hydrolysate-generating enzyme system	DH; protein/nitrogen recovery; MW distribution; solubility; antioxidant or bioactivity readout	[4,5,6,7,8]
Sensory-improved hydrolysates	Bitter peptide persistence; terminal-residue imbalance	Endpoint-refinement protease; exopeptidase; aminopeptidase	Bitterness reduction; small-peptide enrichment; free amino acid release; terminal trimming; sensory-risk prediction	[9,10,11]
Hypoallergenic hydrolysates	Residual epitope-containing peptides	Targeted endoprotease; epitope-removing enzyme combination	Residual antigenicity; IgG binding; peptide mass fingerprinting; epitope-peptide mapping	[12]
Meat tenderization/dairy coagulation	Limited matrix modification; over-proteolysis risk	Matrix-modifying protease; clotting-selective acid protease	Shear force; texture; clotting activity; CA/PA ratio; κ-casein cleavage; curd quality	[7,13,14,15,16]
Beverage stabilization/clarification	Haze-forming protein removal; product-quality preservation	Selective stabilization protease; acid-window clarification protease	Turbidity; haze stability; foam stability; polyphenol retention; storage quality	[22,23]
Keratin side-stream valorization	Insoluble keratin access; disulfide-crosslinked substrate	Keratinase; substrate-opening enzyme system; protease–reductase system	Feather/keratin conversion; soluble protein release; peptide recovery; amino acid release; reducing-agent effect	[17,18,19,20,29,30,31]
Matrix-sensitive processing	Target conversion; non-target matrix preservation	Matrix-selective protease; low-collagenase keratinase	Target removal; collagen preservation; myofibrillar preservation; keratinase/collagenase selectivity; product integrity	[15,32]

**Table 2:** Concise evidence framework for catalyst-defined and endpoint-directed protease studies.

Evaluation Layer	Evidence Focus	Key Evidence	Caution/Consideration	Representative References
Catalyst definition	Active catalyst identity	Host; secretion route; preparation type; purity; mature form; activation state; glycosylation; proteoform verification	Same enzyme name ≠ same catalyst form	[7,34]
Deployment format	Catalyst form in use	Purified enzyme; crude preparation; immobilized enzyme; fermented preparation; loading amount; reuse stability; batch reproducibility	Purified, crude, and immobilized formats ≠ interchangeable evidence	[33,36]
Process translation	Process-condition performance	pH; temperature; reaction time; surfactants; salts; chelators; reducing agents; storage stress; application assay	Buffer activity ≠ process compatibility	[21,24]
Substrate fit	Real-substrate accessibility	Soluble/insoluble substrate; substrate pretreatment; real-matrix assay; substrate-opening requirement; conversion; recovery	Model-substrate activity ≠ real-substrate conversion	[19,20,31]
Product outcome	Endpoint-relevant product quality	DH; MW distribution; LC-MS/MS peptide map; bioactivity; antigenicity; bitterness; turbidity; texture; solubility	High DH or high conversion ≠ high product quality	[5,12,22]
Endpoint refinement	Product-quality correction	Enzyme sequence; enzyme dose; reaction time; terminal trimming; small-peptide enrichment; free amino acid release; optimized stopping point	Additional hydrolysis ≠ automatic improvement	[9,37,38]
Selectivity/matrix preservation	Target-biased proteolysis	Keratinase/collagenase ratio; collagen preservation; myofibrillar preservation; curd integrity; texture preservation; target removal	Stronger degradation ≠ better processing	[15,32]
Metal/cofactor compatibility	Catalytic-state compatibility	Metal-ion requirement; EDTA sensitivity; competing ions; matrix ion composition; cofactor dependence; thermal stabilization by ions	Defined-buffer activity ≠ matrix compatibility	[39,40]
Data-guided design	Hypothesis prioritization	Cleavage prediction; specificity profiling; structure-aware modeling; peptide mapping; product-property prediction; process optimization	Prediction ≠ product-level proof	[41,42,43]
Integrated validation	Product-directed performance	Defined catalyst; relevant process condition; real substrate; endpoint assay; reproducibility; product-quality readout	Single-condition success ≠ broad industrial generality	[12,21,32]

**Table 3:** Class-specific design logic for product-oriented industrial proteases.

Protease Class	Design Logic	Product-Oriented Roles	Key Checkpoints	Ref.
Aspartic proteases	Acid-window endpoint matching	Dairy coagulation; acidic hydrolysis; acidic tenderization; beverage clarification	Acidic pH fit; active/mature form; clotting selectivity; κ-casein cleavage; turbidity, shear-force, or bioactivity endpoint	[7,14,16,23]
Serine proteases	Operating-window and bottleneck-specific engineering	Detergent formulation; cold/thermal processing; keratin conversion; peptide-pool engineering; matrix-selective processing	Process-stress tolerance; surfactant/formulation stability; cold or thermal performance; real-substrate conversion; cleavage-profile alteration; target/non-target selectivity	[4,21,24,28,29,30,32,44,45,46]
Cysteine proteases	Catalyst definition and designability	Food proteolysis; mild tenderization; designed cleavage	Preparation identity; activation requirement; cleavage-site validation; endpoint reproducibility	[34,47,48,49]
Metalloproteases	Metal-state peptide generation and peptide-pool refinement	Neutral protease hydrolysis; primary peptide generation; aminopeptidase debittering; terminal trimming; endpoint correction	Metal-ion dependence; chelator sensitivity; competing-ion effect; matrix compatibility; peptide-pool remodeling; bitterness or small-peptide endpoint	[8,9,39,40]

## Data Availability

No new data were created or analyzed in this study. Data sharing is not applicable to this article.
